# Reactive oxygen species exert opposite effects on Tyr23 phosphorylation of the nuclear and cortical pools of annexin A2

**DOI:** 10.1242/jcs.173195

**Published:** 2016-01-15

**Authors:** Ann Kari Grindheim, Hanne Hollås, Aase M. Raddum, Jaakko Saraste, Anni Vedeler

**Affiliations:** 1 Department of Biomedicine, University of Bergen, Jonas Lies vei 91, Bergen N-5009, Norway; 2 Molecular Imaging Center (MIC), University of Bergen, Jonas Lies vei 91, Bergen N-5009, Norway

**Keywords:** Annexin A2, Tyrosine phosphorylation, Oxidative stress, Nucleus, Cell cortex

## Abstract

Annexin A2 (AnxA2) is a multi-functional and -compartmental protein whose subcellular localisation and functions are tightly regulated by its post-translational modifications. AnxA2 and its Tyr23-phosphorylated form (pTyr23AnxA2) are involved in malignant cell transformation, metastasis and angiogenesis. Here, we show that H_2_O_2_ exerts rapid, simultaneous and opposite effects on the Tyr23 phosphorylation status of AnxA2 in two distinct compartments of rat pheochromocytoma (PC12) cells. Reactive oxygen species induce dephosphorylation of pTyr23AnxA2 located in the PML bodies of the nucleus, whereas AnxA2 associated with F-actin at the cell cortex is Tyr23 phosphorylated. The H_2_O_2_-induced responses in both compartments are transient and the pTyr23AnxA2 accumulating at the cell cortex is subsequently incorporated into vesicles and then released to the extracellular space. Blocking nuclear export by leptomycin B does not affect the nuclear pool of pTyr23AnxA2, but increases the amount of total AnxA2 in this compartment, indicating that the protein might have several functions in the nucleus. These results suggest that Tyr23 phosphorylation can regulate the function of AnxA2 at distinct subcellular sites.

## Abstract (precis)

**Summary:** Reactive oxygen species cause two opposite and transient Tyr23-based modifications of annexin A2; its dephosphorylation in the nucleus and phosphorylation at the cell cortex, resulting in release of the protein in exosomes.

## INTRODUCTION

Annexin A2 (AnxA2) is a multifunctional protein and a member of the annexin superfamily of proteins initially characterised by their Ca^2+^- and lipid-binding properties (Gerke and Moss, 2002; Moss and Morgan, 2004; Gerke et al., 2005; Singh, 2007; Bharadwaj et al., 2013). Additional AnxA2 ligands include the EF-hand protein S100A10, which can form a heterotetramer with AnxA2 (Gerke and Moss, 2002; Gerke et al., 2005), F-actin (Gerke and Weber, 1984; Filipenko and Waisman, 2001), mRNAs (Vedeler and Hollås, 2000; Filipenko et al., 2004; Aukrust et al., 2007; Vedeler et al., 2012) and plasminogen and plasmin (Flood and Hajjar, 2011; Hedhli et al., 2012). The monomeric 39 kDa (36 kDa by SDS-PAGE) AnxA2 consists of two principal domains; a 33 kDa C-terminal core structure folded into a tightly packed α-helical conformation and a unique N-terminal region of 3–4 kDa (Rosengarth and Luecke, 2004).

Post-translational modifications (PTMs) are believed to be important for discrimination between the different functions of AnxA2. Three major phosphorylation sites are located in the N-terminal region: Ser11 (counting the first serine residue as Ser1) (Jost and Gerke, 1996), Ser25 (Gould et al., 1986) and Tyr23 (Glenney and Tack, 1985). AnxA2 is also subjected to other PTMs including N-terminal acetylation of Ser1 (Johnsson et al., 1988), S-glutathiolation of Cys8 (Sullivan et al., 2000), polyubiquitylation (Lauvrak et al., 2005) and sumoylation (Caron et al., 2015).

AnxA2 was originally identified as a major cellular substrate of viral Src (Erikson and Erikson, 1980; Radke et al., 1980) and is also a substrate of other Src family Tyr kinases (Matsuda et al., 2006), as well as receptor Tyr kinases (Rothhut, 1997) such as the insulin receptor kinase (Rescher et al., 2008), leading to a rapid increase in cortical actin (Vedeler et al., 1991). It is involved in the regulation of actin dynamics (de Graauw et al., 2008; Rescher et al., 2008; Hayes and Moss, 2009), as phosphorylation of Tyr23 has been shown to reduce the ability of the AnxA2 heterotetramer to bind and bundle F-actin (Hubaishy et al., 1995; Filipenko and Waisman, 2001), and also induce cell scattering and branch formation (de Graauw et al., 2008). Furthermore, Tyr23 phosphorylation is required for stable binding of AnxA2 to endosomes, transport from early to late endosomes (Morel and Gruenberg, 2009), association of the protein with lipid rafts and multivesicular bodies (MVB), as well as its subsequent localisation to the lumen of exosomes (Valapala and Vishwanatha, 2011). AnxA2 is upregulated in several cancer types (Lokman et al., 2011) and in some cancer cell lines only phosphorylated AnxA2 is detected (Chiang et al., 1996), supporting a role for AnxA2 phosphorylation in cell survival and proliferation (Kumble et al., 1992; Chiang et al., 1996).

AnxA2 is also a redox-sensitive protein (reviewed by Madureira and Waisman, 2013). Cys8 in AnxA2 can be oxidised in response to TNF-α and exogenous H_2_O_2_ (Sullivan et al., 2000; Caplan et al., 2004), and oxidation and/or glutathiolation inhibits its interaction with liposomes and F-actin (Caplan et al., 2004). Oxidised AnxA2 can subsequently be reduced by the thioredoxin system. Therefore, it has been hypothesised that AnxA2 participates in a redox cycle and can degrade several molecules of H_2_O_2_ (Madureira et al., 2011). Accordingly, depletion of AnxA2 led to increased protein oxidation in response to oxidative stress, both at cell and tissue levels (Madureira et al., 2011). Upregulation of AnxA2 in response to H_2_O_2_-induced oxidative stress has been reported for several cell types (Tanaka et al., 2004; Kim et al., 2011; Madureira et al., 2011) and its phosphorylation is also increased under these conditions (Tanaka et al., 2004; Matsuda et al., 2006). A recent study concluded that H_2_O_2_-induced Tyr23 phosphorylation of AnxA2 occurs through the metalloproteinase and sphingolipid pathways (Cinq-Frais et al., 2015). The protein has also been shown to reside in the nucleus, where it contributes to the protection of DNA in cells exposed to H_2_O_2_ and other genotoxic agents (Madureira et al., 2012). Brief temperature stress (heat shock) of endothelial cells can also induce Tyr23 phosphorylation of AnxA2 and its subsequent translocation via an unconventional secretory pathway to the extracellular surface of the plasma membrane (Deora et al., 2004). Moreover, in cultured cone photoreceptor cells, the excitatory neurotransmitter glutamic acid stimulates the translocation of Tyr23-phosphorylated AnxA2 (pTyr23AnxA2) to the cell surface (Valapala et al., 2014). Excessive stimulation (exitotoxicity) of neurons e.g. by glutamic acid can be induced by, as well as induce, oxidative stress (Mattson, 2003), and both exitotoxicity and oxidative stress have been implicated in neurodegenerative diseases. AnxA2 is expressed in subpopulations of neurons found in different brain regions, mainly localised to the intra- or extracellular surface of the plasma membrane and dendritic lipid rafts (Zhao et al., 2004; Zhao and Lu, 2007). It is also upregulated in response to neuronal injury (de la Monte et al., 1995).

Generally, AnxA2 is localised to the cytoplasm, associating with both endomembranes and filamentous (F)-actin (Hayes et al., 2004; Grieve et al., 2012). However, a smaller pool of AnxA2 is also found in the nucleus (Vishwanatha et al., 1992; Eberhard et al., 2001; Liu and Vishwanatha, 2007), where – in addition to DNA protection – it participates in DNA replication by associating with a primer recognition protein complex (Jindal et al., 1991). Further, AnxA2 is involved in transcription by activating the transcription factors STAT3 and STAT6 (Das et al., 2010; Wang et al., 2012). Nuclear fractions contain phosphorylated form(s) of AnxA2, whose appearance in the nucleus depends on the stage of the cell cycle (Liu et al., 2003).

How AnxA2 is targeted to the nucleus remains poorly understood, as the protein does not appear to contain a nuclear localisation signal (NLS). However, because of the presence of a nuclear export signal (NES) in its N-terminus, AnxA2 can be rapidly exported from the nucleus, showing that it acts as a nuclear shuttle protein (Eberhard et al., 2001). It has been hypothesised that the retention of AnxA2 in the nucleus could result from the masking of the NES by ligand interactions and/or phosphorylation (Liu and Vishwanatha, 2007). Although phosphorylation affects both the nuclear import and export of AnxA2 (Eberhard et al., 2001; Liu et al., 2003; Yan et al., 2007; Luo et al., 2008; Grindheim et al., 2014), the detailed mechanisms of these events remain to be elucidated.

Here, we show that nuclear pTyr23AnxA2 is rapidly dephosphorylated in response to oxidative stress, whereas AnxA2 localised to the cell cortex concomitantly undergoes Tyr23 phosphorylation. These are two separate events and do not involve the translocation of AnxA2 from the nucleus to the peripheral cytoplasm. These results suggest that Tyr23 phosphorylation can alter the function of AnxA2 at distinct sub-cellular sites.

## RESULTS

### Localisation of pTyr23AnxA2 under normal and oxidative stress conditions

**Fig. 1.**
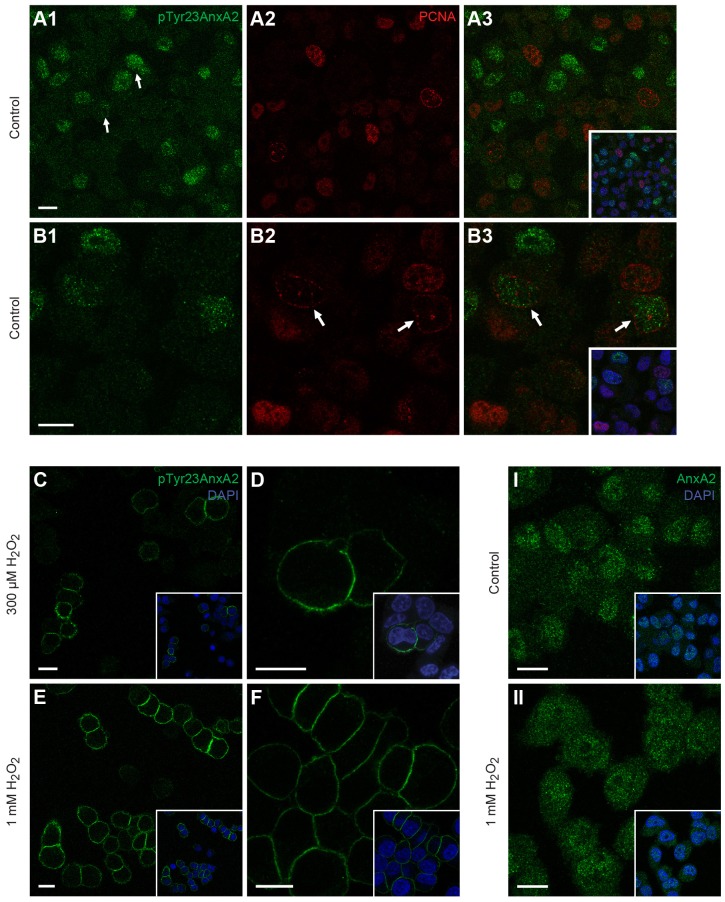
**As a result of H_2_O_2_-induced oxidative stress the cell-cycle-stage-dependent nuclear pool of pTyr23AnxA2 is diminished and replaced by a cortical pool.** PC12 cells were double-stained for immunofluorescence using mono- and polyclonal antibodies against pTyr23AnxA2 (A1,B1, green) and the S-phase marker PCNA (A2,B2, red), respectively. The insets of the merged confocal images (A3,B3) also show DAPI staining (blue) to highlight the nuclei. PC12 cells, either untreated (A1,B1,I), or treated for 15 min with 300 µM (C,D) or 1 mM (E,F,II) H_2_O_2_, were subjected to immunofluorescence staining using specific monoclonal antibodies against pTyr23AnxA2 (C–F, green) or non-phosphorylated AnxA2 (I,II, green). The insets show DNA staining by DAPI (blue). Two nuclei with different signal intensities for pTyr23AnxA2 are indicated by the arrows in A1. The arrows in B2 and B3 indicate cells in late S-phase. Scale bars: 10 µm. This key experiment was repeated more than 15 times and other experiments at least five times.

In accordance with previous reports on the nuclear localisation of AnxA2 (Vishwanatha et al., 1992; Liu et al., 2003; Liu and Vishwanatha, 2007; Madureira et al., 2012), immunofluorescence microscopy of rat pheochromocytoma (PC12) cells, using a monoclonal antibody specifically reacting with pTyr23AnxA2 (Raddum et al., 2013; Bouwman et al., 2014), revealed a punctate nuclear pattern in many cells, in addition to a weaker diffuse cytoplasmic staining (Fig. 1A,B). However, the nuclear signal was highly variable, with only a subset of the cells showing intensive punctate staining (Fig. 1A, compare cells at arrows). Because the nuclear expression of AnxA2 appears to be regulated by the cell cycle (Liu et al., 2003; Wang and Lin, 2014), we next investigated the possible co-appearance of pTyr23AnxA2 and the proliferating cellular nuclear antigen (PCNA), a marker for the S phase. Based on its variable nuclear patterns, the latter also provides a marker for the different sub-phases of the S phase (Celis and Celis, 1985). According to our results, pTyr23AnxA2 is absent from the nucleus during most of the S phase (Fig. 1A,B), but becomes detectable in cells at the late S-phase, which typically contain PCNA-positive speckled structures close to the inner nuclear membrane (see arrows in Fig. 1B2,B3).

Because AnxA2 is known to act in mRNA transport (Mickleburgh et al., 2005; Hollås et al., 2006; Vedeler et al., 2012) and has been suggested to accumulate in the nucleus of cells exposed to oxidative stress (Madureira et al., 2012), we next examined the localisation of pTyr23AnxA2 in PC12 cells after the addition of exogenous H_2_O_2_ to the culture medium. Remarkably, after 15 min incubation in the presence of H_2_O_2_, the nuclear pool of pTyr23AnxA2 was greatly diminished and the phosphorylated protein was found in the cytoplasm, predominantly localised to the cortical region close to the plasma membrane (Fig. 1C–F). By inducing oxidative stress, different concentrations of H_2_O_2_ (300 µm or 1 mM) affected the cortical localisation of pTyr23AnxA2 in a dose-dependent manner (Fig. 1C–F), but not that of non-phosphorylated AnxA2 (Fig. 1I,II).

**Fig. 2.**
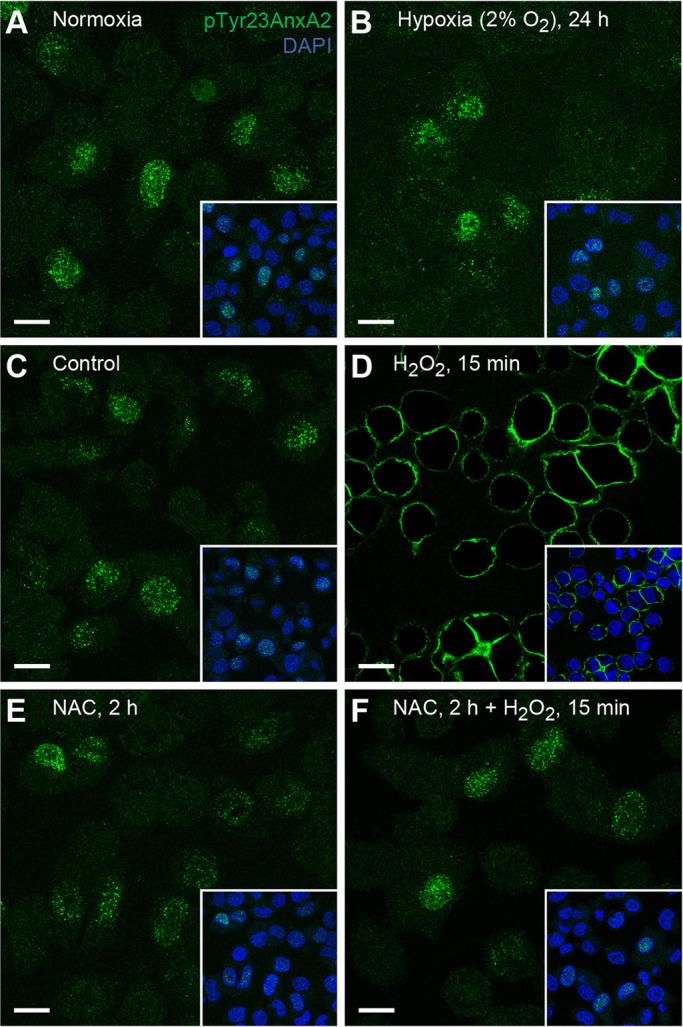
**The distribution of pTyr23AnxA2 is not affected when cells are exposed to either hypoxia or to H_2_O_2_ after their pre-treatment with an antioxidant.** (A,B) PC12 cells were grown for 24 h under control (A, normoxia, 21% O_2_) or hypoxic conditions (B, 2% O_2_). (C–F) Cells were untreated (C); treated for 15 min with 1 mM H_2_O_2_ (D); or incubated for 2 h in the sole presence of 40 mM NAC (E), or with the additional presence of 1 mM H_2_O_2_ during the last 15 min (F). Immunofluorescence staining was carried out using the monoclonal antibody against pTyr23AnxA2 (green). The insets show DNA staining (DAPI, blue) to highlight the nuclei. Scale bars: 10 µm.

To verify the specificity of the observed response, PC12 cells were grown for up to 24 h in low oxygen (2% O_2_) atmosphere to induce hypoxia, another form of oxidative stress. Notably, this treatment had negligible effect on the subcellular localisation of pTyr23AnxA2 (Fig. 2B). Furthermore, when the cells were pre-treated with N-acetyl-cysteine (NAC), an antioxidant and free radical scavenging agent (Aruoma et al., 1989), the localisation of pTyr23AnxA2 was not affected by the subsequently added H_2_O_2_ (Fig. 2F).

**Fig. 3.**
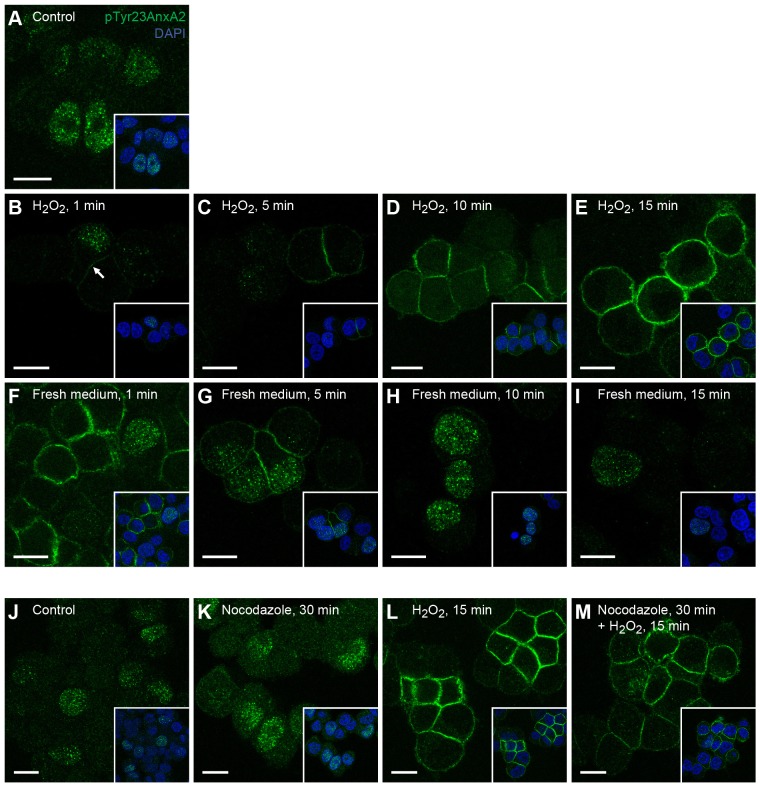
**The effect of H_2_O_2_ on the distribution of pTyr23AnxA2 is rapid and reversible and the appearance of the H_2_O_2_-induced cortical pool of pTyr23AnxA2 does not require intact microtubules.** PC12 cells were untreated (A,J), or treated with 1 mM H_2_O_2_ for 1 min (B), 5 min, (C), 10 min (D) or 15 min (E,L). Subsequently, the medium containing H_2_O_2_ was removed and the cells were incubated in fresh medium for 1 min (F), 5 min, (G), 10 min (H) or 15 min (I). Cells were incubated for 30 min with 10 µg/ml nocodazole (K,M) to depolymerise microtubules; 1 mM H_2_O_2_ was present during the last 15 min of nocodazole treatment (M). Immunofluorescence staining was carried out using the monoclonal pTyr23AnxA2 antibody (green). The merged confocal images (insets) also show DAPI staining (blue) of the nuclei. Arrow in B indicates cortical localisation of pTyr23AnxA2. Scale bars: 10 µm.

The altered distribution of pTyr23AnxA2 in H_2_O_2_-treated cells can be explained in two ways. One possibility is that pTyr23AnxA2 is first exported from the nucleus to the cytoplasm and then transported to the cell cortex. Alternatively, nuclear pTyr23AnxA2 could become dephosphorylated, whereas a separate plasma-membrane-associated pool of AnxA2 undergoes Tyr23 phosphorylation. To distinguish between these possibilities, we followed the process in more detail in a time course experiment where the cells prior to fixation were treated for 1, 5, 10 or 15 min with 1 mM H_2_O_2_ (Fig. 3B–E). As soon as 1 min after H_2_O_2_ addition, pTyr23AnxA2 was detected in the vicinity of the plasma membrane (Fig. 3B, arrow). Subsequently, the cortical signal gradually increased and after 15 min the bulk of pTyr23AnxA2 was found close to the plasma membrane (Fig. 3E), as described above (Fig. 1). In addition, to study the reversibility of the process, H_2_O_2_ was removed after 15 min and the cells were incubated for 1, 5, 10, or 15 min in H_2_O_2_-free medium (Fig. 3F–I). Notably, following H_2_O_2_ wash-out, the cortical signal gradually decreased and pTyr23AnxA2 rapidly reappeared in the nucleus, resuming a localisation pattern similar to that seen in untreated cells within 15 min. The rapid cortical appearance of pTyr23AnxA2 after H_2_O_2_ addition (Fig. 3B), and its presence both in the nucleus and the periphery of the same cells (Fig. 3F,G) indicate that the observed shift in pTyr23AnxA2 distribution is not a result of the relocation of the protein. Accordingly, the disassembly of microtubules by nocodazole had no effect on the cellular distribution of pTyr23AnxA2 either in control or H_2_O_2_-treated cells (Fig. 3K,M), ruling out the involvement of microtubule-dependent transport in this process.

### pTyr23AnxA2 at the plasma membrane initially associates with cortical F-actin in H_2_O_2_-treated cells

**Fig. 4.**
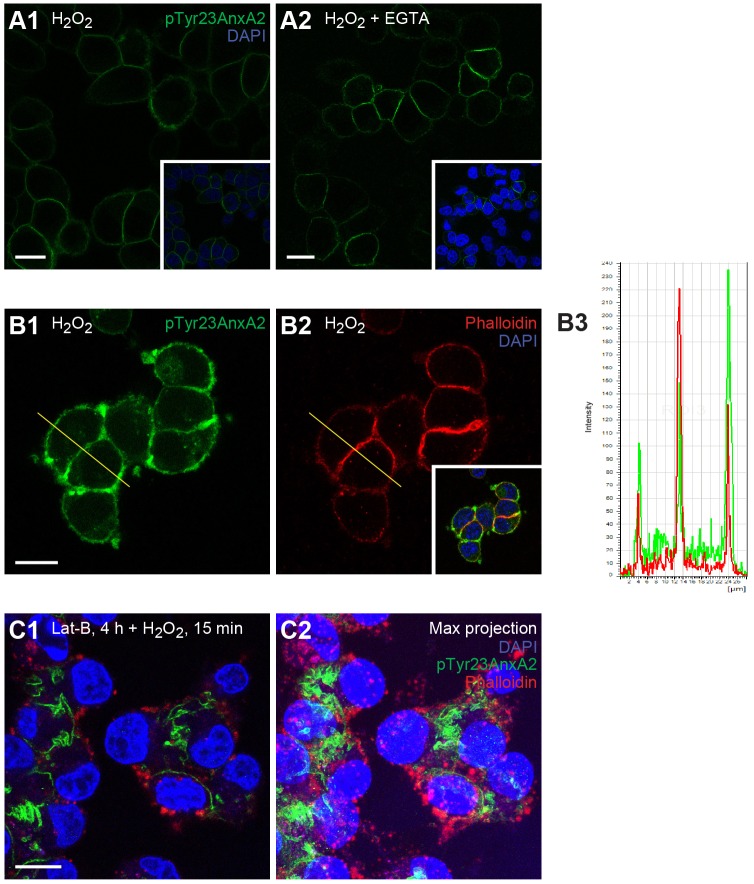
**The H_2_O_2_-induced cortical pool of pTyr23AnxA2 associates with F-actin.** (A1,A2) PC12 cells were treated for 15 min with 1 mM H_2_O_2_ alone (A1), or in the presence of 2 mM EGTA and subsequently washed with H_2_O_2_- and EGTA-containing medium prior to fixation (A2). (B1–C2) Cells were treated with 1 mM H_2_O_2_ alone (B1,B2), or also incubated in the presence of Lat-B to disassemble actin filaments (C1,C2). C1 depicts a single optical section, whereas C2 shows a maximum projection of the same cells to highlight the simultaneous collapse of cortical pTyr23AnxA2 and actin in response to Lat-B. pTyr23AnxA2 (green) and F-actin (red) were detected using a monoclonal antibody or phalloidin, respectively. The merged images (insets in A1,A2,B2; C1,C2) also show the DAPI-stained nuclei (blue). Scale bars: 10 µm. B3 shows the overlap of the fluorescence intensity profiles of pTyr23AnxA2 (green) and phalloidin (red) across the control cell, following the lines indicated in B1 and B2.

Previous studies have shown that Tyr23 phosphorylation of AnxA2 results in the translocation of the protein from the cytoplasm to both the intra- and extracellular sides of the plasma membrane (Deora et al., 2004; Rescher et al., 2008; Grieve et al., 2012). To determine the topology of the plasma-membrane-associated pTyr23AnxA2, the culture medium during the 15 min H_2_O_2_ treatment was supplemented with EGTA, which inhibits the calcium-dependent interaction of AnxA2 with the extracellular matrix (ECM) and results in its release from the extracellular side of the plasma membrane. However, the presence of EGTA in the medium during the incubation and subsequent washing of cells had no effect on the plasma membrane association of pTyr23AnxA2 (Fig. 4A).

Generally, by inducing the accumulation of cortical F-actin, oxidative stress results in a more rounded cell shape. Co-staining of the H_2_O_2_-treated cells (15 min) with anti-pTyr23AnxA2 antibody and phalloidin showed that the phosphorylated protein closely associates with F-actin at the cytoplasmic side of the plasma membrane (Fig. 4B). Treatment of cells with latrunculin B (Lat-B) results in the dissociation of the filament bundles of F-actin and the formation of actin aggregates (Wakatsuki et al., 2001) (Fig. 4C). Following this treatment, pTyr23AnxA2 loses its close association with actin. However, pTyr23AnxA2 is still associated with the structurally altered plasma membrane, particularly at the sites of cell–cell contact (Fig. 4C).

### Oxidative stress causes the incorporation of pTyr23AnxA2 into extracellular vesicles

**Fig. 5.**
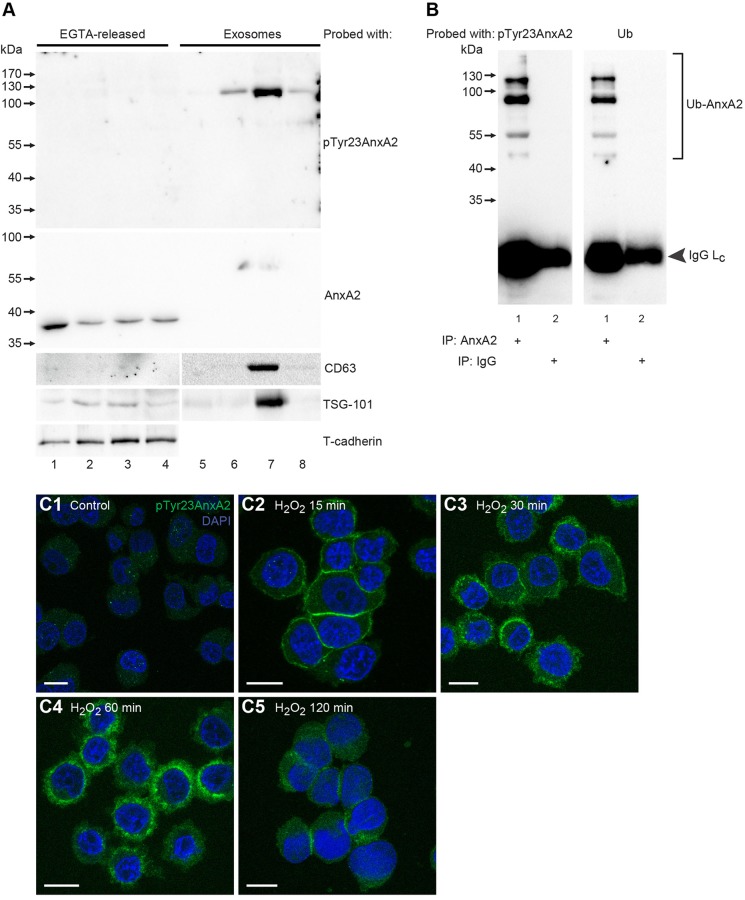
**Incorporation of ubiquitylated pTyr23AnxA2 into extracellular vesicles (exosomes) and decrease in the cortical pool of pTyr23AnxA2 after prolonged treatment of cells with H_2_O_2_.** (A) PC12 cells were grown in exosome-depleted medium in the presence of H_2_O_2_ for 0 min (lanes 1,5), 15 min (lanes 2,6), 1 h (lanes 3,7), or 2 h (lanes 4,8). ECM-bound proteins were released by EGTA (lanes 1–4), whereas extracellular vesicles were isolated from the culture medium by the ExoQuick-TC method (lanes 5–8). 100 µg of protein from the EGTA-released fractions (lanes 1–4) and the control extracellular vesicle fraction (lane 5), or an equal volume of extracellular vesicles from H_2_O_2_-treated cells (lanes 6–8) were separated by 10% SDS-PAGE, transferred to nitro-cellulose membranes and probed with antibodies against pTyr23AnxA2, total AnxA2, CD63, TSG-101 and T-cadherin, as indicated. (B) Following 1 h treatment of PC12 cells with 1 mM H_2_O_2_, proteins (600 µg) present in purified extracellular vesicles were immunoprecipitated (IP) by monoclonal AnxA2 antibodies (lane 1) after pre-clearance of the samples with normal mouse IgG (lane 2). The proteins were subjected to 10% SDS-PAGE and immunoblot analysis using monoclonal antibodies against pTyr23AnxA2 or ubiquitin by loading half of the immunoprecipitation sample on each gel. The bands representing ubiquitylated AnxA2 (square bracket; Ub-AnxA2) and IgG light chain (L_C_, arrowhead) are indicated to the right. (A,B) Following incubation with HRP-conjugated secondary antibodies and the ECL-reagent, the reactive protein bands were detected using the ChemiDoc™ XRS+ molecular imager. Note that the secondary antibody used in B only recognises the IgG light chains. Molecular mass standards are indicated to the left. (C) PC12 cells were untreated (C1), or treated for 15 min (C2), 30 min, (C3), 60 min (C4) or 120 min (C5) with 1 mM H_2_O_2_. The localisation of pTyr23AnxA2, detected using the monoclonal antibody (green), is shown in the merged images, which also display nuclear staining (DAPI, blue). Scale bars: 10 µm.

The above results showed that 15 min after H_2_O_2_ addition, pTyr23AnxA2 is found to be associated with cortical F-actin. Because this phosphorylated form of AnxA2 has previously been localised to endosomes, MVBs, their lumenal exosomes, as well as to the ECM (Deora et al., 2004; Morel and Gruenberg, 2009; Valapala and Vishwanatha, 2011; Zheng et al., 2011), it was of interest to follow the fate of the cortical pTyr23AnxA2 in the H_2_O_2_-treated cells. For this purpose, cells were treated for 15 min, 1 h or 2 h with H_2_O_2_ (1 mM), followed by the isolation of both the ECM proteins (by EGTA treatment) and the extracellular vesicles (including exosomes derived from MVB) from the culture media. Trypan Blue treatment of PC12 cells after H_2_O_2_ treatment showed no effect of the treatment on viability up to 1 h and about 60% viability after 2 h (data not shown). Immunoblotting analysis showed the presence of non-phosphorylated AnxA2 in the ECM of control cells, and its reduction in response to H_2_O_2_, whereas no pTyr23AnxA2 was detected in this fraction (Fig. 5A, lanes 1–4). The high-molecular-mass forms of pTyr23AnxA2 could be detected in the ECM fraction only upon strong over-exposure of the blot (data not shown). By contrast, H_2_O_2_ treatment resulted in the association of pTyr23AnxA2 with extracellular vesicles (Fig. 5A, lanes 5–8), with a peak observed at 1 h (Fig. 5A, lane 7). CD63, a marker for late endosomes, MVBs and exosomes (Andreu and Yáñez-Mó, 2014), was also included in the analysis to distinguish between extracellular vesicle and ECM proteins. The absence of CD63 from the exosomal fraction prior to H_2_O_2_ addition (Fig. 5A, lane 5) indicates the purity of the exosome-depleted medium used, as well as its validity as an extracellular vesicle (‘exosomal’) marker. Tumour susceptibility gene 101 (TSG-101) was also observed in extracellular vesicles with a peak at 1 h after H_2_O_2_ treatment (Fig. 5A, lane 7). Our preliminary results indicate that PC12 cells pre-incubated with exosomes isolated from the medium of H_2_O_2_-treated (1 h) cells become ‘primed’ to better tolerate their subsequent exposure to H_2_O_2_ (1 h), increasing their viability from ∼84% to about ∼93% (data not shown). This observation is in accordance with results obtained with MC/9 cells (Eldh et al., 2010). Also, it was evident that the fluorescent signals corresponding to pTyr23AnxA2 and total AnxA2 at the cell cortex increase in cells preincubated with exosomes and subsequently exposed to H_2_O_2_ (15 min) (data not shown). This finding suggests that the preincubation gives rise to an increased expression of AnxA2, possibly representing an adaptation to oxidative stress (Madureira et al., 2011).

The predominant presence of pTyr23AnxA2 as high-molecular-mass forms indicates that the phosphorylated protein has also undergone other covalent PTMs, e.g. ubiquitylation or sumoylation. Thus, by immunoprecipitation of AnxA2 present in extracellular vesicles and further probing with anti-ubiquitin antibodies, it was shown that pTyr23AnxA2 is indeed ubiquitylated (Fig. 5B). Furthermore, the localisation of pTyr23AnxA2 at the plasma membrane is transient (Fig. 5C1–C5).

### Inhibition of Src kinases inhibits Tyr23 phosphorylation of AnxA2 at the plasma membrane

**Fig. 6.**
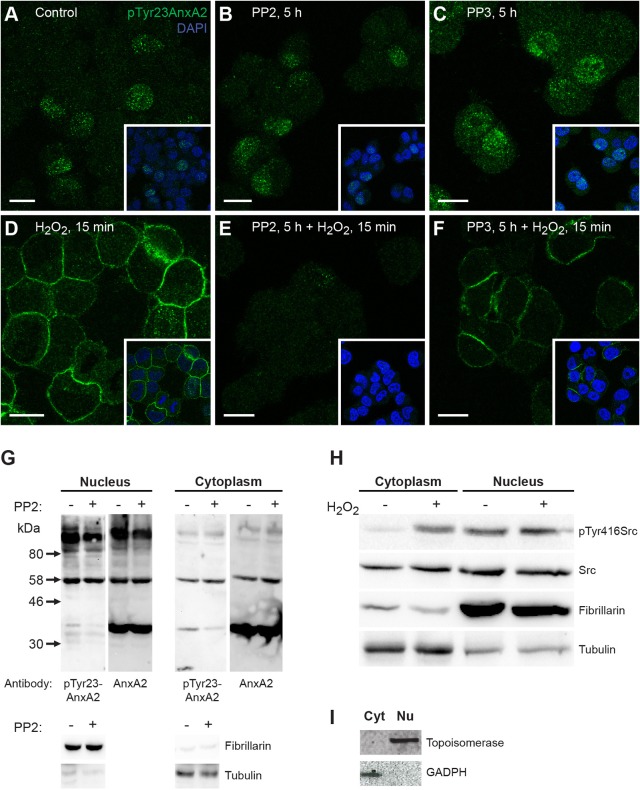
**Cortical AnxA2 is phosphorylated by the Src kinase.** (A–F) Cortical Tyr23 phosphorylation of AnxA2 is blocked by the Src kinase inhibitor PP2. PC12 cells were incubated for 5 h either in control medium (A), or medium containing 20 µM PP2 (B,E) or PP3 (C,F), after which 1 mM H_2_O_2_ was added to the control (D), PP2- (E) and PP3-pre-treated (F) cells for 15 min. The panels show staining of the cells with the antibody against pTyr23AnxA2 (green), whereas the insets represent merged images showing also nuclear staining by DAPI (blue). Scale bars: 10 µm. (G) pTyr23AnxA2 is mainly present as high-molecular-mass forms in the nucleus. Nuclear and cytoplasmic fractions were prepared from control and PP2-treated cells, as indicated. 150 µg of the proteins in each fraction were separated by 10% SDS-PAGE, transferred to nitrocellulose membranes for western blot analysis with monoclonal antibodies against pTyr23AnxA2 or AnxA2 with loading controls for the nucleus (fibrillarin) or cytoplasm (tubulin), as indicated. (H) Src kinase in the cytoplasm is activated during the 15 min treatment with 1 mM H_2_O_2_. Nuclear and cytoplasmic fractions were prepared from control and H_2_O_2_-treated cells, as indicated. 100 µg of the proteins in each fraction were separated by 10% SDS-PAGE, transferred to nitrocellulose membranes for western blot analysis employing antibodies against activated (pTyr416 Src is a marker of Src activation) and total Src. The loading controls for the nucleus (fibrillarin) and cytoplasm (tubulin) are also indicated. (I) Distribution of marker proteins in sub-cellular fractions. 100 μg of proteins from the cytoplasmic (Cyt) or nuclear (Nu) fractions derived from PC12 cells were separated by 10% (w/v) SDS-PAGE and transferred to a nitrocellulose membrane, which was cut in two parts prior to western blot analysis employing antibodies against GAPDH (lower part) or topoisomerase (upper part). Detection of the resulting protein bands was performed using the ChemiDoc™ XRS+ molecular imager after incubation with HRP-complex-conjugated secondary antibodies and ECL-reagent. The arrows to the left of G indicate the protein molecular mass standards.

To further study the Tyr23 phosphorylation of AnxA2, the PC12 cells were treated with PP2, a selective inhibitor of Tyr kinases of the Src-family (Hanke et al., 1996) and subjected to immunofluorescence or cell fractionation and western blot analyses. Pre-treatment with PP2 inhibited the H_2_O_2_-dependent Tyr23 phosphorylation of AnxA2 at the plasma membrane (Fig. 6E). By contrast, in cells pre-treated with PP3, a non-functional analogue of PP2, and subsequently exposed to H_2_O_2,_ pTyr23AnxA2 was still readily detectable at the plasma membrane (Fig. 6F). PP2 did not appear to affect the nuclear pTyr23AnxA2 in control cells that were not exposed to H_2_O_2_, indicating that this pool displays a relatively stable tyrosine phosphorylation status (Fig. 6B,G). In the nuclear and cytoplasmic fractions of PC12 cells total AnxA2 is mainly present in its 39 kDa form, in addition to some high-molecular-mass bands (Fig. 6G). By contrast, pTyr23AnxA2 is predominantly present as high-molecular-mass forms, which appear to be enriched in the nuclear fraction compared with the cytoplasm (Fig. 6G). Moreover, in response to PP2 treatment the amount of the 39 kDa form of pTyr23AnxA2 decreased, whereas its high-molecular-mass forms were largely unaffected (Fig. 6G). The Src kinase in the cytoplasm is activated in response to 15 min H_2_O_2_ treatment (Fig. 6H).

### Nuclear localisation of pTyr23AnxA2

**Fig. 7.**
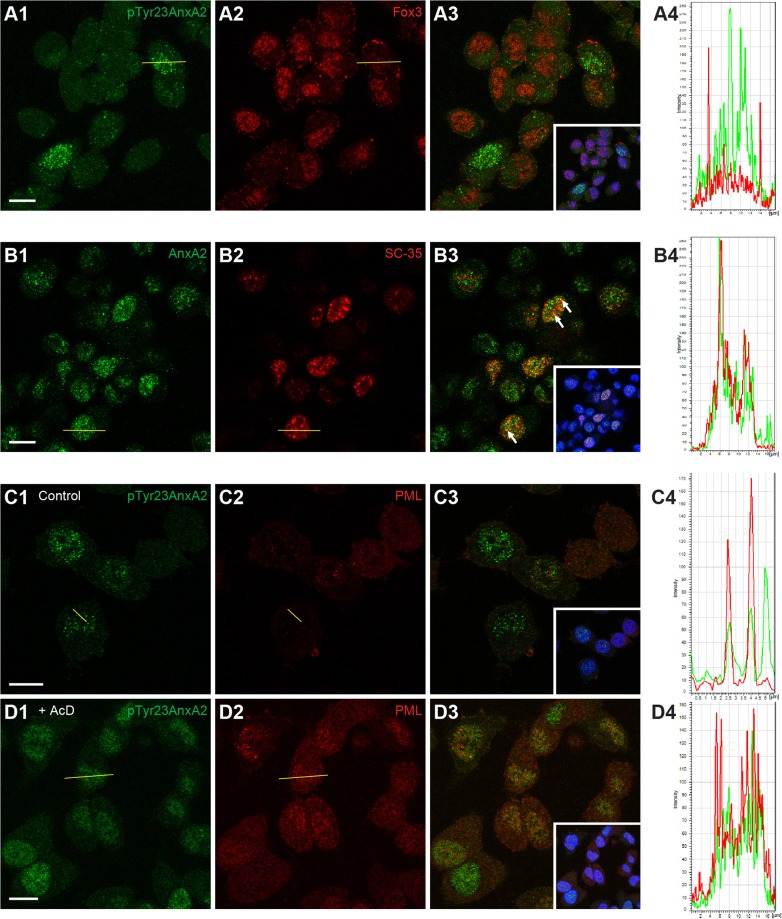
**Nuclear pTyr23AnxA2 is found in PML bodies, but absent in nuclear speckles, whereas AnxA2 is found in SC-35-positive nuclear speckles.** PC12 cells were untreated (A1–A3,B1–B3,C1–C3), or treated for 1 h with 3 μg/ml AcD (D1–D3). The cells were double-stained with monoclonal pTyr23AnxA2 (green) (A1,C1,D1) and polyclonal Fox3 (A2) or PML (C2,D2) (red) antibodies as indicated. Other cells were double-stained with polyclonal AnxA2 (B1, green) and monoclonal SC-35 (B2, red) antibodies. The merged confocal images (insets in A3,B3,C3,D3) also show DAPI staining (blue) of the nuclei. Scale bar: 10 µm. (A4,B4,C4,D4) Fluorescence intensity profiles for the two markers indicated of the cross-sections (from left to right) of control (A–C) and AcD-treated (D) cells indicated by lines on the corresponding images. Arrows in B3 indicate colocalisation of AnxA2 with SC-35-positive nuclear speckles.

Because the nuclear pTyr23AnxA2 displays a predominantly punctate pattern, we first addressed its possible association with nuclear speckles by co-staining the cells with an antibody against the marker protein Fox3. The two proteins showed no colocalisation (Fig. 7A), and interestingly, cells with high nuclear expression of pTyr23AnxA2 typically showed low levels of Fox3, and vice versa (Fig. 7A). Total AnxA2 partially colocalised with another nuclear speckle marker, SC-35 (Fig. 7B, arrows). However, it should be noted that both proteins showed relatively widespread distributions in the nucleus, and their overall staining patterns were dissimilar. Cells with high nuclear expression of total AnxA2 frequently also contained strong SC-35-positive puncta, whereas part of the cell population displayed low expression of both proteins (Fig. 7B). We also compared the localisation of pTyr23AnxA2 with the nucleolar marker fibrillarin, but detected no colocalisation (data not shown). Because an apparent partial colocalisation of AnxA2 and SC-35 was observed, overall transcription was inhibited by actinomycin D (AcD), resulting in the formation of RNA-containing bodies that typically contain the histone γ-H2AX (Mischo et al., 2005). Whereas very little overlap was observed in untreated cells, the AcD-treatment resulted in apparent partial colocalisation of γ-H2AX and pTyr23AnxA2 (data not shown). However, the two proteins display distinct patterns in many cells and their expression levels are frequently inversely related. AcD might also cause DNA damage and γ-H2AX has been used as a marker for this event (Sharma et al., 2012). Thus, we reduced the AcD concentration to avoid DNA damage and investigated the possible association of pTyr23AnxA2 with the promyelocytic leukaemia (PML) bodies. Notably, colocalisation was observed both in untreated (Fig. 7C) and, in particular, AcD-treated cells (Fig. 7D). We observed that following AcD treatment both PML and pTyr23AnxA2 displayed a relatively diffuse nuclear distribution with small foci, in accordance with the previously reported formation of PML microbodies in response to AcD (Eskiw et al., 2004). Also, the overall patterns of pTyr23AnxA2 and PML were more similar than observed for the other nuclear markers tested.

**Fig. 8.**
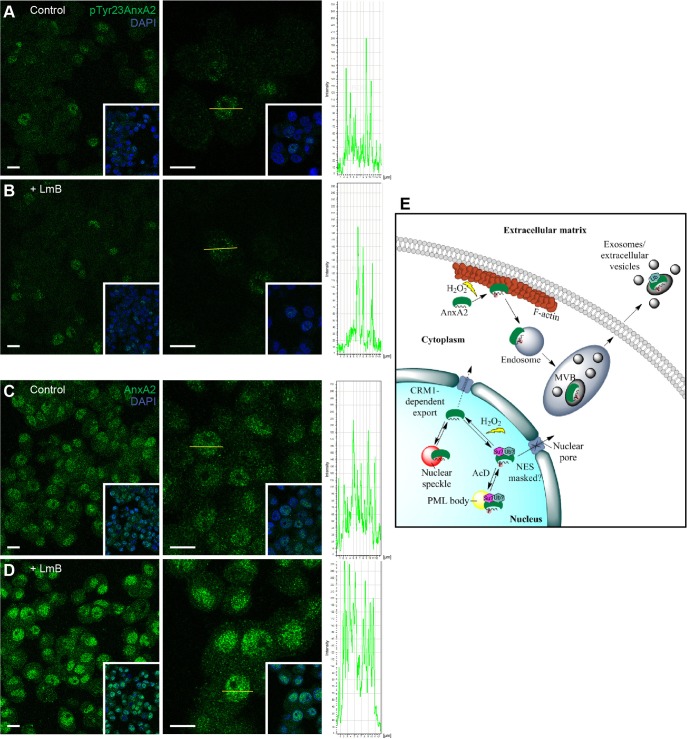
**Nuclear export of AnxA2, but not of pTyr23AnxA2, is blocked by LmB.** PC12 cells were untreated (A,C) or treated for 2 h with LmB (B,D), followed by immunofluorescence staining using monoclonal pTyr23AnxA2 (A,B) or polyclonal AnxA2 (C,D) antibodies. The middle panels show higher magnification images with lines indicating cross-sections of nuclei, with the left-to-right orientation of the lines corresponding to the fluorescence intensity profiles shown to the right. The insets include DAPI staining (blue) to verify the position of the nuclei. Scale bars: 10 µm. (E) A schematic model of the transient Tyr23 AnxA2 phosphorylation events occurring at two distinct sites in response to H_2_O_2_. Upon exposure to H_2_O_2_, nuclear Tyr23-phosphorylated AnxA2 in PML bodies rapidly dephosphorylates and might become associated with SC-35-positive nuclear speckles. AcD increases the localisation of pTyr23AnxA2 in PML bodies suggesting a role in transcription. Note that the pTyr23AnxA2 form is not exported from the nucleus (via the CRM1-dependent pathway). At the same time cortical AnxA2 is Tyr23 phosphorylated, associates with actin and subsequently with endosomes and multivesicular bodies (MVB) and is exported out of the cell in the lumen of exosomes. pTyr23AnxA2 appears to be ubiquitylated and/or sumoylated.

PML bodies are considered as dynamic multifunctional nuclear compartments. They have been implicated in transcription, apoptosis and DNA-damage and/or might represent a storage site for inactive proteins within the nucleus (Lallemand-Breitenbach and de The, 2010). To further address the proposed role of nuclear pTyr23AnxA2, and AnxA2 in general, in mRNA export from the nucleus, we employed leptomycin B (LmB) to inhibit CMR1-mediated nuclear export (Liu et al., 2003; Kazami et al., 2014). Although the nuclear signal of pTyr23AnxA2 remains unaffected by the treatment (Fig. 8A,B), the amount of total AnxA2 evidently increases in the nucleus (Fig. 8C,D). It should be noted that more cells display AnxA2-positive than pTyr23AnxA2-positive nuclei (Fig. 8A–D), possibly indicating more than one function of AnxA2 in the nucleus.

## DISCUSSION

### Role of AnxA2 in oxidative stress response

H_2_O_2_ and other reactive oxygen species (ROS) are highly reactive compounds capable of damaging a variety of macromolecules, including proteins, lipids and DNA. They have been implicated in various disorders, such as Alzheimer's and Parkinson's disease, diabetes and cancer. Besides being a by-product of the mitochondrial respiratory chain, low levels of H_2_O_2_ can also be produced by different cells and tissues in response to various ligands, such as certain growth factors, insulin, cytokines and angiotensin II. H_2_O_2_ participates in receptor signalling, cell growth, proliferation, apoptosis and senescence, and is associated with increased pTyr-dependent signalling (Rhee et al., 2000b; Chiarugi and Cirri, 2003; Rhee et al., 2003; Lambeth, 2004; Milton and Sweeney, 2012). Furthermore, H_2_O_2_ can also induce fully reversible protein oxidation. For example, it can induce reversible glutathiolation of Cys residues, as exemplified by Cys8 of AnxA2 (Caplan et al., 2004), which is involved in several aspects of cellular protection against oxidative stress. Transcription factors, such as nuclear factor κ-B, activator-protein I, hypoxia-inducible factor and p53, as well as the p21/Ras family of proto-oncogenes contain reactive cysteine residues that can be oxidised by H_2_O_2_ (Chiarugi and Cirri, 2003). AnxA2 has been suggested to function in DNA repair (Madureira et al., 2012), as an antioxidant by being a substrate of thioredoxin (Madureira et al., 2011) and its depletion increases protein oxidation and ROS levels, as well as cell death in response to oxidative stress or ROS-producing drugs. Long-term treatment of 293T and MCF7 cells with 100 µM H_2_O_2_ results in upregulation of AnxA2. Upregulation of AnxA2 via VEGF and ERK signalling is also caused by hypoxia in osteoblastic and cervical epithelial cells (Denko et al., 2000; Genetos et al., 2010).

In this study, the final concentration of H_2_O_2_ in the culture medium was 1 mM, i.e. the same concentration as has recently been applied to investigate its effect on nuclear AnxA2 (Madureira et al., 2012). H_2_O_2_ readily traverses the plasma membrane, reaching an estimated intracellular concentration that is 7–10 times lower than the extracellular concentration (reviewed by Stone and Yang, 2006). In our case this would predict an intracellular H_2_O_2_ concentration of ∼100–140 µM, which causes oxidative stress. Our key observation was that when PC12 cells are exposed to H_2_O_2_ the nuclear signal of pTyr23AnxA2 rapidly disappears and, concomitantly, a new pool of pTyr23AnxA2 appears at the cytoplasmic side of the plasma membrane (Fig. 1). A similar effect was observed in HeLa cells (data not shown). This change in subcellular distribution of pTyr23AnxA2 in PC12 cells could also be induced by lower concentrations of H_2_O_2_ (50 µM H_2_O_2_, predicted to result in an intracellular concentration of ∼5 µM), but required longer incubation times, as the Tyr23 phosphorylation of cortical AnxA2 was observed 2 h after the addition of H_2_O_2_ (data not shown). When exogenously added H_2_O_2_ is taken up by cells, its intracellular concentration rapidly decreases as a result of degradation by glutathione peroxidases, catalases and thioredoxin (Hashida et al., 2002; Gülden et al., 2010). Consequently, the stimulatory effects on tyrosine phosphorylation return rapidly to basal levels (Lee et al., 1998; Rhee et al., 2000b). Taking into account the effects of H_2_O_2_ on pTyr levels and its rapid cellular degradation, as well as to avoid transcriptional effects, we decided to employ a higher concentration of H_2_O_2_ (1 mM) and short incubation times to investigate in detail how H_2_O_2_ affects the subcellular distribution of pTyr23AnxA2.

As previously reported in the case of nuclear accumulation of total AnxA2 (Madureira et al., 2012), the dose-dependent, rapid and reversible effects of H_2_O_2_ on cellular distribution of pTyr23AnxA2 could be blocked by the antioxidant and free radical scavenger NAC (Fig. 2E,F), indicating specificity. In addition, the cellular distribution of pTyr23AnxA2 was not affected by a 15 min (data not shown) or even 24 h (Fig. 2A,B) exposure of cells to hypoxia (2% O_2_), indicating that the altered Tyr23 phosphorylation pattern of AnxA2 is caused by ROS, rather than being part of a general stress response to hypoxia. Translocation of the heterotetrameric AnxA2–S100A10 complex to the plasma membrane has been shown to take place after 30 min of hypoxia, and is caused by changes in intracellular pH (Monastyrskaya et al., 2008).

### Rapid oxidative-stress-induced Tyr23 phosphorylation of AnxA2 at the plasma membrane

The disappearance of pTyr23AnxA2 in the nucleus concomitantly with the appearance of pTyr23AnxA2 at the plasma membrane involves two separate pools of AnxA2. Several findings support this conclusion. Firstly, the appearance of pTyr23AnxA2 at the plasma membrane takes place after a 1 min incubation of cells with H_2_O_2_ (Fig. 3). Secondly, nuclear and cortical pTyr23AnxA2 can be detected in the same cells during a short incubation with H_2_O_2_ (Fig. 3), and simultaneous inhibition of nuclear export with LmB does not affect the appearance of cortical pTyr23AnxA2 (data not shown). Thirdly, following H_2_O_2_ wash-out pTyr23AnxA2 rapidly resumes the localisation pattern seen in untreated cells (Fig. 3). Finally, the cortical appearance of pTyr23AnxA2 does not require intact microtubules (Fig. 3), but is blocked by pre-treatment of the cells with the Src family inhibitor PP2 (Fig. 6) and the distribution of non-phosphorylated AnxA2 does not change in H_2_O_2_-treated PC12 cells (Fig. 1I,II). H_2_O_2_ treatment activates the Src kinase in the cytoplasm (Fig. 6), suggesting the involvement of this kinase in the pTyr23 phosphorylation of cortical AnxA2.

Other stimuli can also induce Tyr23 phosphorylation of AnxA2 and its association with the cell periphery. Insulin leads to rapid Tyr23 phosphorylation of a cortical pool of AnxA2 associated with the accumulated actin filaments. This event is mediated by the insulin receptor and not affected by PP2 (Rescher et al., 2008). The association of AnxA2 with both insulin and insulin-like growth factor-1 receptors in PC12 cells is reduced upon insulin stimulation, which also increases the secretion of AnxA2 (Zhao et al., 2003). Stress conditions such as hypoxia and heat shock can induce Tyr23 phosphorylation of AnxA2 and its cell surface expression in human umbilical vein endothelial cells (HUVECs) (Deora et al., 2004; Huang et al., 2011). In line with these data, we previously observed that anti-pTyr23AnxA2 was the most potent antibody in inhibiting network formation by HUVECs in a co-culture system mimicking several features of angiogenesis (Raddum et al., 2013). Because Tyr23 phosphorylation of AnxA2 and its association with the plasma membrane appear to be involved in malignant cell transformation, metastasis and angiogenesis (de Graauw et al., 2008; Mohammad et al., 2008; Zheng et al., 2011), the detailed characterisation of these events is of importance.

H_2_O_2_ treatment increases the intracellular Ca^2+^ concentration (Wang and Joseph, 2000), triggering a conformational change in AnxA2 and increasing its membrane binding (Rosengarth and Luecke, 2004). Moreover, plasma membrane association of AnxA2 increases the accessibility of the Tyr23 residue and lowers the Ca^2+^ concentration required for phosphorylation, thereby enhancing its modification by the Src kinase (Bellagamba et al., 1997). Higher intracellular Ca^2+^ levels and pTyr23 phosphorylation lead to the association of pTyr23AnxA2 with lipid rafts, its transport via endosomes, incorporation into the lumenal exosomes of MVBs and delivery to the extracellular space (Valapala and Vishwanatha, 2011).

To determine whether the pTyr23AnxA2 appearing at the periphery of H_2_O_2_-treated cells is located at the intra- or extracellular side of the plasma membrane, extracellular Ca^2+^-binding proteins (including AnxA2) were released by EGTA after 15 min exposure of PC12 cells to oxidative stress. Evidently, pTyr23AnxA2 was not released under these conditions (Fig. 4A). Further, cortical pTyr23AnxA2 overlaps with F-actin (Fig. 4B). The disintegration of actin filaments with Lat-B apparently leads to an enrichment of pTyr23AnxA2 at sites of cell–cell contact (Fig. 4C), where it also overlapped with β-catenin (data not shown). This is in accordance with the results showing that AnxA2 is recruited to VE-cadherin- and β-catenin-containing cell–cell junctions in confluent HUVECs, and that its interaction with these complexes is independent of F-actin (Heyraud et al., 2008).

When the incubation time with 1 mM H_2_O_2_ was extended to 2 h, the plasma-membrane-associated pool of pTyr23AnxA2 appeared to decrease considerably (Fig. 5C). Therefore, we investigated the appearance of the protein in the ECM or extracellular vesicles at 0, 15 min, 1 h and 2 h after the exposure of cells to H_2_O_2_ and obtained evidence for its release in exosome-like vesicles (Raposo and Stoorvogel, 2013), with maximum release taking place about 1 h after H_2_O_2_ addition (Fig. 5A). The similar patterns of CD63 and TSG-101 localisation compared with pTyr23AnxA2 after H_2_O_2_ treatment suggest that the latter might be associated with exosomes. It has been shown that oxidative stress increases the release of exosomes (Hedlund et al., 2011) and subsequently mediates protective messages in mouse mast cells (MC/9) (Eldh et al., 2010).

Interestingly, immunoblotting reveals that the main bands of pTyr23AnxA2 are about 90 and 120 kDa, indicating additional covalent PTM(s), most likely ubiquitylation and/or sumoylation. Thus, we performed IP of AnxA2 present in exosomes and found that pTyr23AnxA2 is indeed ubiquitylated (Fig. 5B). We and others have previously shown that AnxA2, like its closest relative annexin A1 (AnxA1), is ubiquitylated (Lauvrak et al., 2005; Hirata et al., 2010; Deng et al., 2012). This modification is stable and does not appear to target the protein for degradation (Lauvrak et al., 2005). Furthermore, polyubiquitylated proteins are enriched in the lumen of exosomes, as compared with total cell lysates (Buschow et al., 2005). Thus, specific PTMs of extracellular vesicle proteins could be linked to intercellular transmission of oxidative stress conditions, which is known to take place via exosomes (Eldh et al., 2010; de Jong et al., 2012).

### Localisation of pTyr23AnxA2 in the nucleus

Cell fractionation showed the nuclear enrichment of pTyr23AnxA2 and immunofluorescence microscopy further revealed its typical punctate pattern in the nucleus of a subset of PC12 cells. In accordance with previous studies (Liu et al., 2003), pTyrAnxA2 was predominantly undetectable in the nucleus during S phase of the cell cycle (Fig. 1A,B). Furthermore, pTyr23AnxA2 was mainly present in the nucleus as high-molecular-mass forms, indicating that it had undergone covalent modifications, e.g. ubiquitylation and/or sumoylation. Upon treatment with PP2, the minor 39 kDa form of pTyr23AnxA2 was decreased, whereas the nuclear high-molecular-mass forms were largely unaffected (Fig. 6), indicating their relatively slow phosphorylation–dephosphorylation turnover. Covalent PTMs could affect the accessibility of pTyr23AnxA2 to modifying enzymes, explaining the stable phosphorylation status of its high-molecular-mass forms. It should be mentioned that besides exosomes, the enrichment of polyubiquitylated and sumoylated proteins has been reported also for nuclear PML bodies (Lallemand-Breitenbach and de The, 2010; Pankiv et al., 2010).

The nuclear pTyr23AnxA2 is mainly localised to the interchromatin space, but excluded from the nucleolus (Fig. 7). In addition to residing in strongly fluorescent puncta, the protein also displays a more diffuse distribution throughout the nucleoplasm. The punctate pattern is similar to that displayed by nuclear speckles or interchromatin granules, which contain proteins involved in pre-mRNA processing (Spector and Lamond, 2011). However, pTyr23AnxA2 does not seem to associate with nuclear speckles, whereas non-phosphorylated AnxA2 showed partial colocalisation with their marker protein SC-35. This is consistent with the involvement of AnxA2 in both transcription – via its interaction with the transcription factors STAT3 and STAT6 (Das et al., 2010; Wang et al., 2012) – and mRNA transport (Mickleburgh et al., 2005; Vedeler et al., 2012). Interestingly, nuclear phosphatidylinositol(4,5)-biphosphate, an AnxA2 ligand (Hayes et al., 2009), also colocalises to SC-35-positive nuclear structures (Osborne et al., 2001).

We observed that inhibition of CMR1-mediated export by LmB leads to nuclear accumulation of non-phosphorylated AnxA2, but does not affect nuclear pTyr23AnxA2 (Fig. 8), indicating Tyr23 phosphorylation ‘tags’ AnxA2 for a specific function in the nucleus. Thus, it is possible that the nuclear pool of non-phosphorylated AnxA2 might have a role in mRNA transport. By contrast, pTyr23AnxA2 is not affected by this treatment, indicating that phosphorylation is connected with a specific nuclear function of AnxA2.

Oxidative stress by H_2_O_2_ also results in the upregulation of AnxA1 and its redistribution to the perinuclear cytoplasm and the nucleus (Rhee et al., 2000a). However, it remains unknown whether these changes affect the phosphorylation status of AnxA1. Still another annexin, AnxA10, has been found in the nucleus and was shown to relocate in doxorubicin- or AcD-treated cells to dark nucleolar caps, where it associates with proteins normally found in paraspeckles (Quiskamp et al., 2014).

Interestingly, we observed that pTyr23AnxA2 is at least partly localised to nuclear PML bodies (Fig. 7). Moreover, nuclear pTyr23AnxA2 is mainly absent during the S-phase of cell cycle, but becomes detectable during the late S-phase (Fig. 1) when heterochromatin, a minor part of the genome that is typically found around the centromere and telomeres and contains noncoding, highly repetitive satellite DNA sequences (John, 1988), is replicated (Leach et al., 2000). AnxA2 interacts with SMARCA3, a protein involved in ATP-dependent chromatin remodelling (Oh et al., 2013) that might also be involved in DNA repair (Gong et al., 2013). The presence of pTyr23AnxA2 in nuclear PML bodies, which have been proposed to function in heterochromatin remodelling during G2 (Luciani et al., 2006), suggest its involvement in this process. Interestingly, PML bodies are dynamic and recruit multiple proteins that have been described as oxidative-stress-responsible sumoylation factories (Sahin et al., 2014). They are believed to be key players in the organisation of compartments and/or domains within the nucleus (Bernardi and Pandolfi, 2007). All the functions of PML bodies might not have been unraveled yet, but they appear to participate in such processes as DNA repair, apoptosis and/or senescence and transcription (Lallemand-Breitenbach and de The, 2010). SC-35-containing nuclear domains have been implicated in the coupled steps of mRNA metabolism and transport (Shopland et al., 2002). Thus, it might be that nuclear AnxA2 has a functional role in many diverse processes including RNA transport and DNA repair as previously suggested (Mickleburgh et al., 2005; Madureira et al., 2012; Vedeler et al., 2012).

In conclusion, H_2_O_2_ exerts two simultaneous, but spatially distinct, effects on Tyr23-based modification of AnxA2: (i) dephosphorylation of pTyr23AnxA2 in the nucleus and (ii) Tyr23 phosphorylation of another pool of AnxA2 located close to the plasma membrane. Both are specific ROS-mediated responses caused by the exposure of cells to oxidative stress (Fig. 8E).

## MATERIALS AND METHODS

### Cell cultures and drug treatments

The rat pheochromocytoma (PC12) cells representing a readily adherent sub-clone derived from the original PC12 cell line (Greene and Tischler, 1976) were kindly provided by Prof. Eyvind Rødahl, Haukeland Hospital, Bergen, Norway. Cells were recently authenticated and routinely tested for contamination. As described previously (Grindheim et al., 2014), the cells were routinely cultured at 37°C in a humidified atmosphere of 21% O_2_ supplemented with 5% CO_2_, except for the hypoxic experiments where the O_2_ level was 2%. As indicated, cells were treated with 300 μM or 1 mM H_2_O_2_ (Sigma) for 15 min (if not indicated otherwise); 40 mM N-acetyl-cysteine (NAC) (Sigma) for 2 h (the pH of the medium was adjusted before addition to the cells); 10 μg/ml nocodazole (Sigma) for 30 min; 2 mM ethylene glycol tetraacetic acid (EGTA; Sigma) for 15 min; 20 or 50 µM 4-amino-5-(4-chlorophenyl)-7-(dimethylethyl)pyrazolo[3,4-d] (PP2; Calbiochem) and 4-amino-1-phenyl-1H-pyrazolo[3,4-d]pyrimidine (PP3; Calbiochem) for 30 min or 5 h; 10 μM latrunculin B (Lat-B; Sigma-Aldrich) for 4 h; 3 or 9 μg/ml actinomycin D (AcD; Sigma-Aldrich) for 1 h or 5 h, respectively; and 20 nM Leptomycin B (LmB; Sigma-Aldrich) for 2 h.

### Immunofluorescence

PC12 cells were grown on poly-L-Lys-coated glass coverslips and treated as indicated. Cells were fixed, permeabilised and blocked as described previously (Grindheim et al., 2014), prior to staining with primary antibodies against pTyr23AnxA2 (sc-135753, Santa Cruz Biotechnologies, 1:20), AnxA2 (ab41803, Abcam, 1:250), Fox3 (ABN51, Millipore, 1:100), SC-35 (NB100-1774, Novus Biologicals, 1:500), PCNA (Ab18197, Abcam, 1:200), PML (Sc-5621, Santa Cruz Biotechnologies, 1:100), Fibrillarin (C13C3, Cell Signaling, 1:100) and γ-H2A.X (07-164, Millipore, 1:200) as indicated. The bound primary antibodies were detected using appropriate DyLight-488- or DyLight-594-conjugated Fab_2_ fragments (Jackson ImmunoResearch Laboratories, 1:50). F-actin was detected directly by Alexa-Fluor-594-conjugated Phalloidin (Life Technologies). The coverslips were inverted and mounted on objective glasses on a small drop of Vectashield mounting medium containing 4′,6-diamino-2′-phenylindole (DAPI) (Vector Laboratories). Confocal imaging was performed using a Leica SP5 AOBS confocal laser scanning microscope equipped with 405 diode, argon and helium neon lasers (Leica Microsystems, Germany). Optical sections were obtained using the 63×/1.4 NA HCX Plan-Apochromat oil-immersion objective (Leica), ∼1 Airy unit pinhole aperture and appropriate filter combinations. Confocal images were obtained in Leica Application Suite (LAS) AF. Figures were made in Adobe Illustrator CS5.1 except Fig. 8E, which was made in ChemBioDraw Ultra 14.0 (PerkinElmer).

### Cell fractionation

Nuclear and cytoplasmic fractions were prepared from PC12 cells using the NE-PER Nuclear and Cytoplasmic Extraction Kit (Thermo Scientific). GAPDH and topoisomerase were highly enriched in the cytoplasmic and nuclear fractions, respectively (Fig. 6I), indicating the relative purity of the fractions obtained with this method. For the isolation of extracellular vesicles, PC12 cells were grown in medium supplemented with exosome-depleted serum (System Biosciences) and extracellular vesicles were purified from the medium with the ExoQuick kit (System Biosciences) following the manufacturer's instructions as described (Zhu et al., 2014).

### Immunoprecipitation with anti-AnxA2 antibodies

Proteins (600 µg) present in extracellular vesicles purified from the medium after their treatment for 1 h with H_2_O_2_ were immunoprecipitated using monoclonal anti-AnxA2 antibodies (2.75 µg, BD Biosciences, 610069) coupled to protein G-Sepharose following pre-clearance of the vesicles by protein G-Sepharose-coupled normal mouse IgG. Immunoprecipitation was performed in NET-buffer (50 mM Tris-HCl pH 7.4, 150 mM KCl, 0.05% Triton X-100 and 0.2 mM CaCl_2_) overnight at 4°C, after which the beads were sedimented for 5 min by centrifugation at 800×***g*** at 4°C. After extensive washing with NET-buffer, the immune complexes were eluted in SDS-PAGE sample buffer.

### SDS-PAGE and western blot analysis

SDS-PAGE was performed using 10% (w/v) gels and the proteins were transferred onto nitrocellulose membranes (0.2 µm pore size) by overnight blotting performed at 150 Vh. Total AnxA2 or its Tyr23-phosphorylated form were detected using monoclonal antibodies directed against AnxA2 (610069, BD Biosciences, 1:1000) or pTyr23AnxA2 (sc-135753, Santa Cruz Biotechnologies, 1:200) (Spijkers-Hagelstein et al., 2013). CD63 and T-cadherin were detected using rabbit polyclonal antibodies (EXOAB-CD63A-1, System Biosciences, 1:500 and sc-7940, Santa Cruz, 1:1000); ubiquitin was detected using mouse monoclonal antibodies [13-1600 (Ubi-1), Invitrogen, 1:1000]. Activated Src (pTyr416 Src), Src and fibrillarin were detected by monoclonal rabbit antibodies from Cell Signaling at a 1:1000 dilution (D49G4, 32G6 and C13C3, respectively). Subsequently, HRP-labelled secondary anti-rabbit or anti-mouse antibodies (170-6515, Bio-Rad, 1:5000 and 115-035-174, Jackson ImmunoResearch Laboratories, 1:5000) were used. The reactive protein bands were visualised using the Supersignal West Pico- or Femto Chemiluminescent Substrate kits (Pierce).
